# Trabecular Bone Score Significantly Influences Treatment Decisions in Secondary Osteoporosis

**DOI:** 10.3390/jcm12124147

**Published:** 2023-06-20

**Authors:** Leith Al-Hashimi, Jens Klotsche, Sarah Ohrndorf, Timo Gaber, Paula Hoff

**Affiliations:** 1 MVZ Endokrinologikum Berlin am Gendarmenmarkt, 10117 Berlin, Germany; leith.al-hashimi@amedes-group.com; 2 Corporate Member of Freie Universität Berlin and Humboldt Universität zu Berlin, Department of Rheumatology and Clinical Immunology, Universitätsmedizin Berlin, 10117 Berlin, Germany; sarah.ohrndorf@charite.de (S.O.); timo.gaber@charite.de (T.G.); 3 German Rheumatism Research Centre (DRFZ) Berlin, a Leibniz Institute, 10117 Berlin, Germany; jens.klotsche@drfz.de

**Keywords:** secondary osteoporosis, bone mineral density, dual-energy X-ray absorptiometry (DXA), trabecular bone score/TBS, fragility fracture

## Abstract

The trabecular bone score (TBS) can be determined in addition to the Dual Energy X-ray Absorptiometry (DXA) for bone mineral density (BMD) measurement to diagnose, evaluate, and stratify bone loss and decide on appropriate treatment in patients at risk. Especially in patients with secondary osteoporosis, TBS detects restricted bone quality. To investigate the influence of an additional evaluation of TBS on patients’ treatment strategy decisions, we enrolled 292 patients, with a high proportion of patients with secondary osteoporosis, from one outpatient unit over one year. Patients eligible for BMD measurement had the option to opt-in for TBS measurement. We analyzed demographic data, leading diagnoses, bone metabolism parameters, and results of BMD and TBS measurements. More than 90% of patients consented to TBS measurement. TBS measurement influenced the decision in approximately 40% of patients with a treatment indication for anti-osteoporotic drugs. We demonstrate that depending on the underlying disease/risk spectrum, 21–25.5% of patients had an unremarkable BMD measurement with poor bone quality shown in the TBS measurement. In patients with secondary osteoporosis, the use of TBS supplementary to DXA seems useful to better assess fracture risk and, thus, to initiate therapy for osteoporosis in these patients in time.

## 1. Introduction

Osteoporosis is characterized by reduced bone mineral density and impaired bone microarchitecture. This leads to an increased risk of fracture [1,2,3,4,5,6,7]. Typical osteoporotic fractures include vertebral body, proximal femur, and distal radius fractures. These are associated with significant morbidity and mortality and result in high socioeconomic costs [1,2,3,4,5,6,7,8].

In 1994, the World Health Organization (WHO) defined the terms osteopenia and osteoporosis based on a dual-energy X-ray absorptiometry (DXA) measurement of bone mineral density (BMD). Two regions are considered, the lumbar spine and the femur. A patient with a BMD standard deviation (SD) of at least 2.5 below the young (20–29 years old) female adult (T-score £ −2.5) is defined as having osteoporosis. A BMD with a T-score between −2.5 and −1.0 is determined as osteopenia [7,9]. This definition does not consider the medical history of a patient. Depending on the DXA measurement, patients may be scored as osteopenic or even as having normal BMD, even though they have experienced osteoporotic vertebral fractures requiring osteoporosis treatment. Therefore, other methods are required to evaluate risk factors such as bone quality, including TBS or DXA-based 3-dimensional (3D) modeling (3D-DXA) [10,11]. There are national and international guidelines for the prophylaxis and therapy of osteoporosis, such as the German guideline [7] or the recommendations of the International Society for Clinical Densitometry (ISCD) [12,13], in which an additional TBS measurement is already taken into account. However, the solitary use of TBS to derive a therapy recommendation is not recommended. In contrast to the German guideline, a good TBS can also reduce fracture risk, according to the ISCD [12,13]. 

Osteoporosis can generally occur as primary osteoporosis, which results from aging and decreasing sex hormone levels [14]. Secondary osteoporosis, on the other hand, occurs due to other primary diseases [15,16]. These primary diseases resulting in secondary osteoporosis include numerous rheumatic disorders such as rheumatoid arthritis or spondylarthritis, endocrinological diseases such as hyperparathyroidism, or diseases requiring glucocorticoid therapy [4,5,17,18,19,20]. Other risk factors include alcohol consumption and smoking [21,22]. Therefore, typically patients presenting with primary osteoporosis are older [14]. In contrast, secondary osteoporosis can occur in older and younger patients, such as premenopausal women and young men [14]. Glucocortocoid-induced osteoporosis is the most common secondary osteoporosis [14]. More than half of fractures in the general population occur in patients without osteoporosis as defined by the T score, leading us to the broader definition of osteoporosis [23,24]. An additional analysis of the bone microarchitecture/quality can be helpful here.

Thus, evaluating the trabecular bone score (TBS) is important, especially in patients with secondary osteoporosis. The Manitoba study shows that some patients escape risk evaluation [25]. Therefore, methods that additionally contribute to risk evaluation are important. Alternatives such as quantitative CT (QCT) and high-resolution peripheral quantitative CT (HR-pQCT) measure volumetric BMD and distinguish the information from trabecular and cortical bone [26]. A non-ionizing technique is based on ultrasound with Radiofrequency Echographic Multi-Spectrometry (REMS) [26,27]. The next level could comprise DXA-based 3-dimensional (3D) modeling (3D-DXA) [10,11]. A 3D-Shaper software reconstructs the femur’s 3D shape and density distribution from 2D DXA scans. Nevertheless, TBS measurement is most easily and increasingly available as part of the usual DXA measurement. DXA measurement is recommended in many international and German guidelines and is widely available; it is associated with a very low radiation dose and low costs [26]. TBS evaluation requires extra software, which must be purchased. TBS measures the variation of the grey pixels in the spine DXA scan. It reflects the bone microarchitecture and, thus, the bone quality [28,29]. TBS gives additional information to the DXA scan, predicts the fracture risk independently from BMD measurement, and can be integrated into fracture risk evaluation [7,30]. 

We wanted to test it in a real-life setting at a large medical outpatient center for Endocrinology and Rheumatology. The aim of our study was to evaluate how often, in our special patient population, the additional TBS measurement determines the therapy decision and to compare the DXA and TBS usefulness in a cohort of patients with a high proportion of cases of secondary osteoporosis.

## 2. Materials and Methods

### 2.1. Study Design and Patient Involvement

Patients receiving longitudinal care at the public outpatient clinic of the MVZ Endokrinologikum (outpatient clinic specialized in endocrinology, endocrinological gynecology, osteology, and endocrinology, located at Friedrichstr. 76, 10117 Berlin, Germany) and undergoing a DXA measurement as part of their usual care were included in our study. Data from 06/2019–05/2020 were analyzed retrospectively. A total of 292 patients received osteodensitometry, of whom, 267 also received TBS measurement. Three patients were excluded due to a measurement that could not be technically evaluated. A total of 264 patients were further analyzed. This project was approved by the Ethics Committee of Berliner Ärztekammer, Friedrichstraße 16, 10969 Berlin, Germany (protocol code Eth-KB 07/20).

### 2.2. Eligibility Criteria

Inclusion criteria include (1) age of at least 18 years, (2) diagnosis of an inflammatory rheumatic disease or an endocrine diseases associated with bone loss, (3) current or previous treatment with GC, (4) eligibility for osteoporosis diagnostics as recommended by the Dachverband Osteologie (http://dv-osteologie.org; for full description, see https://dv-osteologie.org/osteoporose-leitlinien, accessed on 1 January 2021), and (5) patients who have consented to TBS measurement

Key exclusion criteria: (1) pregnancy or lactation, (2) inability to provide informed consent for any reason, and (3) patients who declined TBS measurement.

### 2.3. Data Collection 

Data were obtained by routine laboratory assessment, patient survey, and examination. Data collected from each patient are summarized in the following Table 1:

### 2.4. Bone Densitometry

Bone mineral density (BMD) was assessed at the lumbar spine and left (if not possible, the right) proximal femur using dual X-ray absorptiometry (DXA).

All participants were scanned by Lunar Prodigy bone densitometers (GE Medical Systems Lunar Corporation, Madison, WI, USA) per manufacturer recommendations and analyzed with enCORE Software. 

The DXA scan results are presented as T-scores. DXA T-scores > −1.0 were classified as normal, ≤−1.0 to >−2.5 were classified as osteopenic (for analysis normal and osteopenic values were summarized as non-pathological), and T-score ≤ −2.5 osteoporotic (pathological). 

The TBS is calculated from the data available in the DXA scan using the TBS iNsight software [31]. TBS values > 1.23 was classified as non-pathological, TBS values < 1.23 were classified as pathological. The software database allows the calculation of the corresponding T- and Z-scores.

### 2.5. Statistical Analysis

Standard descriptive statistics were performed to report the distribution of sociodemographic and clinical parameters. The DXA and TBS measurements were compared by a Welch test between males and females in different patient groups. The concordance of non-pathological and pathological findings among DXA and TBS was determined by calculating the rate of absolute agreement and the kappa coefficient. Linear regression analyses were performed to determine the association of DXA with TBS, vitamin D, and the bone remodeling markers in univariate and multivariable analysis. Standardized beta coefficients were calculated to compare the strengths of association between parameters. The strength of association according to the standardized beta can be categorized into small (<0.1), medium (0.1 to 0.3), and large (>0.3) for continuously distributed variables. Statistical analyses were performed using STATA 12.1.

## 3. Results

### 3.1. High Patient Acceptance for TBS Measurement and the Strong Influence of TBS on Therapy Decision

Osteodensitometry was performed on 264 patients with an increased risk of developing osteoporosis or with an already diagnosed osteoporosis who were willing to receive a TBS measurement according to the inclusion/exclusion criteria. A total of 109 patients had an indication for antiosteoporotic therapy in addition to vitamin D supplementation, e.g., with bisphosphonates. A total of 65 of these patients (59.6%) exhibited an indication for an antiosteoporotic therapy independently of the TBS measurement. The TBS value influenced the therapy decision in 44 patients (40.4%).

### 3.2. Demographic and Laboratory Data

We analyzed sociodemographic and clinical characteristics, leading diagnoses, bone metabolism parameters, and the results of BMD and TBS measurements.

Overall, more women (81.5%) than men (18.5%) underwent osteodensitometry with the additional TBS measurement (Table 2). Regarding the age distribution, 28% of the patients studied were ≤55 years old, 46% were between 55 and 75 years old, and 26% were 76 years old or older.

Patients were divided into four subgroups depending on the underlying disease and/or ongoing medication. The underlying disease and/or ongoing medication or, as in subgroup 4, the age or the osteoporotic fracture were the indications for the DXA/TBS measurements (Table 2):

1. Patients with ongoing glucocorticoid therapy (due to inflammatory rheumatic disease, endocrinological disease, or other indications such as COPD),
2. Patients with only inflammatory rheumatic diseases (e.g., rheumatoid arthritis),
3. Patients with only endocrine disease (e.g., primary hyperparathyroidisms), and
4. Patients with the indication for bone examination: higher age or an osteoporotic fracture that had occurred.

Subgroups 1–3 represent patients with a high probability of secondary osteoporosis, and subgroup 4 represents elderly patients with primary osteoporosis.

The mean height, weight, and BMI did not differ significantly among the four groups. The mean height was 166 cm, body weight 69.3 kg, and BMI 25.2 kg/m^2^. Females were, by mean, smaller than males, as expected in the overall population.

### 3.3. Comparison of DXA and TBS Measurements in Different Patient Groups

When comparing the T-score of the DXA measurement in the lumbar spine L1–L4 region and the T-score of the TBS measurement, it was noticeable that in all four groups, the T-score of the DXA measurements was higher than the T-score of the TBS measurement, in both men and women (Table 3).

### 3.4. Do DXA and TBS Match in the Classification of Bone Status?

We decided to group patients with normal BMD (DXA T-Score > −1.0) and osteopenic patients (DXA T-Score ≤ −1.0–>−2.5) according to the WHO definition as non-pathological. Although patients with normal or osteopenic BMD might have significant fracture risk, it is difficult to evaluate by the BMD measurement. Especially in this low-risk group, according to the BMD, we wanted to evaluate the potential discrepancy to the risk shown by the impaired bone quality evaluation via TBS. According to the WHO definition, the patients with osteoporosis with a BMD T-Score ≤ −2.5 were assigned as patients with pathological BMD. The same scheme was used for the TBS Score, TBS reflecting normal trabecular microarchitecture (>1.31) or only marginally impaired microarchitecture (1.31–1.23) were defined as non-pathological. TBS < 1.23 showing impaired trabecular microarchitecture was defined as pathologic [31]. Thus, the strongly impaired bone detected by pathological BMD or TBS could be analyzed.

**Figure 1:**
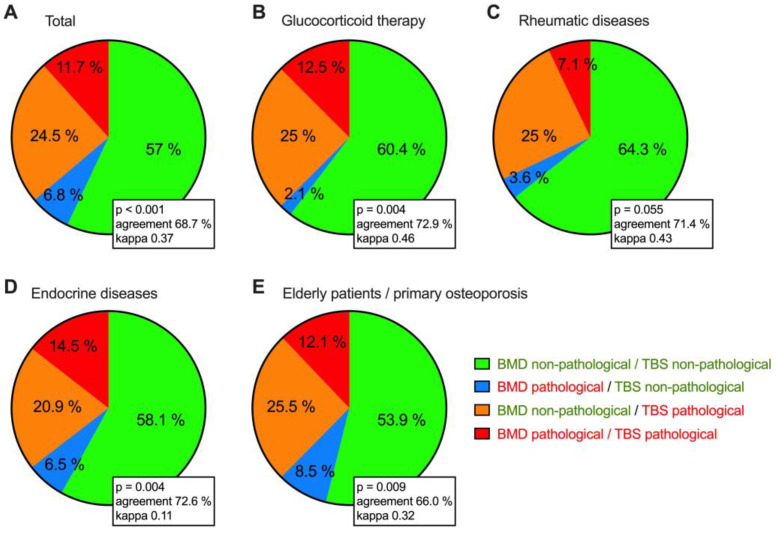
Comparison of conformance between DXA and TBS measurements in different patient groups ((**A**) total patients, (**B**) patients with glucocorticoid therapy, (**C**) patients with rheumatic diseases, (**D**) patients with endocrine diseases, (**E**) elderly patients/patients with primary osteoporosis). *p* value as determined by Chi^2^ test to compare the distributions of BMD and TBS findings, kappa statistics for concordance between BMD and TBS findings. Pathologicals values are labeled in red writing, no-pathological in green writing.

In 57% of the total collective, DXA, and TBS values consistently indicated a non-pathological bone status. In 11.7%, DXA and TBS values were concordantly in the pathological range. In 24.5% of the patients, the DXA values were in the non-pathological range, while the TBS readings were in the pathological range. This group of patients thus shows that the additional TBS measurement is necessary in order to avoid underestimating their fracture risk. In a small percentage (6.8%), the DXA values were in the pathological range, while the TBS readings were in the non-pathological range (Figure 1).

Regarding every single subgroup, it was noticeable that in about a quarter of all four groups, the DXA values were in the non-pathological range, while the TBS values were in the pathological range. Furthermore, we could show that in all four groups, the proportion of patients in whom the DXA measurements were in the pathological range while, at the same time, the TBS measurements were in the non-pathological range, was low (≤8.5%) (Figure 1).

The comparative analysis of the classification according to either DXA measurement or TBS measurement showed little concordance between the results of the two approaches. Focusing on the individual subgroups, we again observed only a low concordance.

### 3.5. Association Analysis of DXA T-Scores, TBS, Vitamin D, and the Bone Remodeling Markers

Our work showed a correlation between the T-score and Z-score of the TBS measurement on the one hand and the T-score and Z-score of the DXA measurement on the other hand. 

It was remarkable that the R-square between the T-score of the DXA measurements and the T-score of the TBS measurements was higher than that between the T-score of the DXA measurement and the Z-score of the TBS measurement. This suggests that the T-score of the TBS measurement may predict the T-score of the DXA measurement better than the TBS scan’s Z-score (Table 4).

Furthermore, in our work, no statistically significant correlation could be shown between the laboratory values on the one hand, and the TBS and DXA measurements on the other hand. This lack of correlation was particularly evident in the univariate analysis. Thus, based on the level and depth of the bone remodeling markers (alkaline bone phosphatase, deoxypyridinoline) and vitamin D concentration, no association can be shown for the TBS or the DXA measurements. 

The multivariant analysis showed a significant association between vitamin D level and the DXA T-score of the total femur and TBS value. However, this association was not detectable in the femoral neck region or the spine region (L1–L4) (Table 4).

## 4. Discussion

Osteoporosis is known to cause a decrease in BMD and a deterioration in bone microarchitecture [7]. BMD can be measured using DXA measurement, based on the absorption and attenuation of X-rays passing through the different body compartments. The microarchitecture of the bone can be quantitatively analyzed by assessing the TBS, which is based on the density and distribution of the individual pixels of the DXA images from the spine [32].

Of note, TBS measurement is independent of degenerative bone abnormalities and is, therefore, better able to detect bone health problems. Although DXA measurement remains the gold standard for diagnosing osteoporosis and assessing fracture risk, it is also accepted that it alone underestimates fracture risk in many pathologies associated secondarily with osteoporosis [6,33,34,35,36,37,38]. TBS measurement as an adjunct to DXA measurement increases the accuracy of fracture risk assessment. In addition, TBS determination is superior to DXA measurements in several types of secondary osteoporosis [6,33,34,35,36,37]. Our investigation showed that in more than 40% of patients with an indication for anti-osteoporotic therapy according to the German DVO guideline [7], the therapy decision was determined by the additional TBS measurement. This is a high percentage, which might be explained by the specialization of our outpatient clinic, as we treat many patients with underlying rheumatic or endocrine diseases or other risk factors for osteoporosis. The evaluation of our data has confirmed the need to use this additional TBS method, especially in our patient collective. Our result is consistent with the results of the Manitoba study, which demonstrated that the TBS is an independent predictor of osteoporosis-related fractures and that measuring it in combination with DXA may provide an advantage over using a DXA investigation alone [25]. 

TBS measurement offers many advantages, but also has limitations. TBS measurement is always coupled to the BMD assessment and requires additional cost-intensive software [34]. Not every osteodensitometry device is equipped with this software, limiting the availability of TBS acquisition. Moreover, as of right now, TBS is only available for the lumbar spine region [34]. Secondly, TBS is only assessable and evaluable for patients with a BMI between 15–37 kg/m^2^ [39]. Higher BMI could correlate with worse TBS scores [40,41]. In particular, our data emphasize the use of DXA and TBS measurement in daily routine, especially in patients with a high probability of secondary osteoporosis. Although the diagnostic benefits of TBS are becoming clearer, there are much less data available to assess treatment response. However, since the increase in TBS value is greater with osteoanabolic therapy compared to antiresorptive therapy [42,43], TBS is also likely to assess therapy response. TBS could therefore play an increasing role in therapy evaluation in the future. Currently, TBS is also being further developed for the hip region and thus represents a new possibility, as shown in [44]. It remains to be seen to what extent other methods such as DXA-based 3-dimensional (3D) modeling (3D-DXA) will become routine in the future [10,11].

The reason for the strikingly lower TBS T-scores compared with the DXA T-scores in our work can be partly explained by ageing-related degenerative abnormalities, since approximately 71.7% of the patients were older than 56 years and 25.7% were even older than 76 years (see Table 1). Brinjikji et al. (2015) systematically reviewed articles that commonly dealt with computed tomography and magnetic resonance imaging data from 3110 individuals focusing on age-related asymptomatic degenerative changes of the spine [45]. They found that the prevalence of asymptomatic spinal degeneration doubles or even triples between the 20th and 80th years of life. Thus, a high proportion of degenerative changes of the spine can also be assumed in the patients recorded by us, even without the corresponding diagnoses already documented. In the case of degenerative changes, the DXA values often falsely prove to be too high [46]. In contrast, TBS measurement is considered to be largely stable and unaffected by degenerative changes, which has been demonstrated in several publications in the past [47,48,49].

The decrease in the quality of the bone microarchitecture with increasing age, which we ultimately record via TBS measurement, has also already been demonstrated histologically in a paper by Mosekilde L. et al. [50]. To this end, they took bone biopsies from the lumbar vertebra L3 of 23 healthy individuals aged between 15–87 years and examined them histologically. It was found that with increasing age, the thickness of the horizontal bone trabeculae decreased and both the horizontal and vertical distances between the bone trabeculae increased. Only the thickness of the vertical bone trabeculae remained constant [50]. 

It has been shown that in patients with type 1 or type 2 diabetes mellitus or acromegaly, DXA T-scores were higher than in healthy subjects, although the actual fracture risk in patients with these diseases is significantly higher [51]. However, TBS measurements were lower in these patient groups than in healthy subjects, leading to the conclusion that TBS measurement is a better predictor of fracture risk than DXA measurement in these patients [51]. Similar results are also shown in patients with Cushing’s syndrome, hyperthyroidism, and primary hyperparathyroidism; here, the TBS T-scores were also always lower than the DXA T-scores [6]. In patients with hyperparathyroidism in particular, vertebral body fractures occur frequently, regardless of whether a risk is recorded by bone densitometry. In contrast, trabecular bone score is a very good predictor of the occurrence of vertebral fractures in patients with primary hyperparathyroidism [52]. 

There is good evidence that TBS can provide additional benefits, not only in our internal medicine practice areas but also in surgery [49]. These colleagues have shown that preoperative assessment of bone status in patients requiring cervical or lumbar spondylodesis identifies more patients with impaired bone stability by DXA measurement supplemented by TBS measurement than by DXA measurement alone. They suggested that these results might be used to predict perioperative complications such as pseudarthrosis, pedicle screw loosening, and fractures of adjacent segments, because timely initiation of anti-osteoporosis drug therapy reduced or prevented these complications [49].

We did not find any statistically significant correlation between the laboratory bone turn over markers and the TBS and DXA measurements in the univariate analysis. The multivariant analysis showed a significant association between vitamin D level and DXA T-score of the total femur and TBS value. However, this association was not detectable in the femoral neck region or in the spine region (Table 3). The positive effects of balanced vitamin D levels on bone health are indisputable [3,18,53,54]. Thus, our results showing an association between DXA T-score and vitamin D are not surprising. We may not have been able to determine any other association with bone turnover markers because of the limited number of patients included in our study; however, at least in our cohort, we confirmed the association of total femoral T score with vitamin D levels, leading us to conclude that while markers of bone turnover are very useful in a clinical setting for monitoring therapy, they are not essential for treatment decisions.

Finally, we have visualized the concordance of pathological versus non-pathological BMD and TBS in Figure 1. In 57% of the total cohort, we observed non-pathological values consistently for both measurements, and in 11.7%, both values are in the pathological region. Interestingly, we find a small group of patients with pathological BMD but non-pathological TBS. This might be best explained by ongoing therapy, which might affect TBS more quickly than BMD. However, most importantly, we have identified a group of about 25% of our patients with non-pathologic BMD but pathologic TBS. 

These findings again confirm the importance of including TBS in the evaluation of osteoporosis, at least in medical centers treating diseases associated with secondary osteoporosis [18,55,56,57,58,59].

## 5. Conclusions

This work has shown that TBS measurement is well accepted by patients in addition to DXA measurement. The use of this examination can help physicians decide on drug therapy for osteoporosis. Especially in patients with secondary osteoporosis, the use of TBS as a supplement to DXA seems to be helpful and useful, as it can better assess the fracture risk, and thus, can help to initiate timely drug therapy for osteoporosis in these patients. 

## Figures and Tables

**Table 1:** Data collected from each patient (by medical record evaluation and measurements).

Type of Parameter	Parameter
Demographics and general information	Age, sex, height, weight, BMI
Underlying disease	Inflammatory rheumatic diseases (rheumatoid arthritis, psoriatic arthritis, spondyloarthritis, connective tissue diseases, vasculitis, polymyalgia rheumatica); endocrine diseases (primary hyperthyroidism, hypogonadism, crushing’s disease, hypophysitis, hyperthyreosis, Diabetes mellitus); Other diseases (monoclonal gammopathy of undetermined significance (MGUS), therapy with aromatas inhibitors after breast cancer).
Antiosteoporotic medication	Vitamin D supplementation, treatment with raloxifene, bisphosphonates (oral, i.v.), denosumab, teriparatide
Bone-relevant laboratory parameters	Vitamin D levels, bone alkaline phosphatase, deoxypyridinoline, C-telopeptide crosslaps of type I collagen (CTX-1), osteocalcin

BMI = body mass index.

**Table 2:** Sociodemographic and clinical characteristics of the included patients.

		Total n = 265		Glucocorticoid Therapy n = 48		Rheumatic Diseases n = 56		Endocrine Diseases n = 62		Elderly Patients/Primary Osteoporosis n = 141	
	N		%	N	%	N	%	N	%	N	%
Sex											
Male	49		18.5	12	25.0	10	17.9	13	21.0	23	16.3
Female	216		81.5	36	75.0	46	82.1	49	79.0	118	83.7
Age, mean (Sd)		62.6 (14.9)		63.8 (13.2)		65.0 (12.6)		55.8 (18.8)		64.3 (13.0)	
≤55 years	75		28.3	11	22.9	10	17.9	31	50.0	33	23.4
56–75 years	122		46.0	24	50.0	31	55.4	15	24.2	74	52.5
76+ years	68		25.7	13	27.1	15	26.8	16	25.8	34	24.1
Height in m, mean (SD)		1.66 (0.09)		1.65 (0.10)		1.65 (0.10)		1.68 (0.10)		1.65 (0.08)	
Weight in kg, mean (SD)		69.3 (14.7)		72.5 (15.6)		71.1 (14.4)		69.2 (16.2)		68.5 (14.2)	
BMI in kg/m^2^, mean (SD)		25.2 (4.8)		26.5 (5.2)		26.3 (4.9)		24.3 (4.7)		25.0 (4.6)	
Bone alkaline phosphatase U/L mean (SD), normal range 4.7–27.1		18.7 (8.3)		19.0 (10.0)		18.8 (8.8)		19.2 (8.7)		18.4 (7.5)	
25-OH-Vitamin D nmol/L, mean (SD), normal range 19.0–139.0		78.9 (67.5)		76.9 (33.3)		79.3 (30.0)		64.2 (25.8)		85.5 (89.8)	
Deoxypyridnoline nmol/mmol, mean (SD), normal range 3.0–7.4		7.4 (5.9)		6.9 (2.6)		7.3 (2.3)		9.7 (12.8)		6.9 (3.4)	

BMI = body mass index, kg = kilogram, m = meter, SD = standard deviation.

**Table 3:** Comparison of DXA and TBS measurements in different patient subgroups.

		Total N = 265		Male N = 49		Female N = 216		
		Mean (SD)	Median	Mean (SD)	Median	Mean (SD)	Median	*p* Value
Total								
	T-score L1–L4	−1.11 (1.60)	−1.25	−1.44 (1.53)	−1.30	−1.04 (1.61)	−1.20	0.107
	T-score femoral neck	−1.30 (1.11)	−1.40	−1.83 (1.09)	−2.00	−1.17 (1.09)	−1.30	<0.001
	T-score femur	−1.23 (1.16)	−1.30	−1.57 (1.12)	−1.65	−1.15 (1.15)	−1.30	0.021
	TBS T-score	−2.23 (1.43)	−2.10	−1.82 (1.49)	−1.70	−2.32 (1.40)	−2.20	0.034
	TBS Z-score	−0.27 (1.25)	−0.20	−0.63 (1.31)	−0.50	−0.18 (1.22)	−0.10	0.045
	TBS value	1.25 (0.13)	1.27	1.25 (0.15)	1.26	1.25 (0.12)	1.27	0.774
Glucocorticoid therapy								
	T-score L1–L4	−1.02 (1.59)	−1.20	−1.28 (1.50)	−1.20	−0.92 (1.64)	−1.20	0.496
	T-score femoral neck	−1.25 (1.06)	−1.40	−1.46 (1.02)	−1.90	−1.18 (1.07)	−1.30	0.434
	T-score femur	−1.08 (0.96)	−1.20	−1.20 (1.01)	−1.30	−1.05 (0.96)	−1.00	0.666
	TBS T-score	−2.36 (1.53)	−2.15	−1.58 (1.73)	−1.30	−2.62 (1.39)	−2.30	0.079
	TBS Z-score	−0.51 (1.43)	−0.20	−0.75 (1.34)	−0.50	−0.44 (1.47)	−0.15	0.537
	TBS value	1.24 (0.14)	1.27	1.28 (0.19)	1.31	1.23 (0.12)	1.26	0.440
Rheumatic diseases								
	T-score L1–L4	−0.62 (1.64)	−0.80	−1.11 (1.88)	−0.90	−0.50 (1.58)	−0.80	0.364
	T-score femoral neck	−1.10 (1.09)	−1.20	−1.61 (1.28)	−2.00	−1.00 (1.04)	−1.05	0.205
	T-Score femur	−0.92 (1.08)	−1.00	−1.22 (0.96)	−1.30	−0.86 (1.10)	−1.00	0.335
	TBS T-score	−2.11 (1.49)	−2.15	−1.88 (1.94)	−2.00	−2.16 (1.39)	−2.15	0.669
	TBS Z score	−0.13 (1.32)	0.00	−0.59 (1.41)	−0.20	−0.03 (1.30)	0.05	0.298
	TBS value	1.27 (0.14)	1.27	1.26 (0.20)	1.25	1.27 (0.13)	1.27	0.935
Endocrine diseases								
	T-score L1–L4	−1.04 (1.78)	−1.00	−1.28 (1.51)	−1.40	−0.98 (1.86)	−0.95	0.556
	T-score femoral neck	−1.12 (1.32)	−1.30	−1.47 (1.19)	−1.50	−1.02 (1.34)	−1.30	0.253
	T-score femur	−1.19 (1.37)	−1.20	−1.31 (1.29)	−1.10	−1.16 (1.40)	−1.30	0.721
	TBS T-score	−2.23 (1.58)	−2.00	−1.24 (0.99)	−1.00	−2.50 (1.60)	−2.20	0.001
	TBS Z-score	−0.62 (1.23)	−0.35	−0.26 (0.91)	−0.10	−0.72 (1.30)	−0.40	0.233
	TBS value	1.26 (0.14)	1.28	1.32 (0.11)	1.34	1.24 (0.14)	1.27	0.053
Elderly patients/primary osteoporosis								
	T-score L1–L4	−1.31 (1.48)	−1.40	−1.75 (1.47)	−1.60	−1.22 (1.47)	−1.40	0.136
	T-score femoral neck	−1.42 (1.02)	−1.60	−2.12 (0.98)	−2.10	−1.28 (0.98)	−1.40	0.001
	T-score femur	−1.34 (1.08)	−1.45	−1.83 (1.10)	−1.80	−1.24 (1.06)	−1.30	0.024
	TBS T-score	−2.26 (1.35)	−2.10	−2.07 (1.54)	−1.80	−2.30 (1.31)	−2.20	0.519
	TBS Z-score	−0.19 (1.21)	−0.20	−0.75 (1.48)	−0.60	−0.06 (1.11)	−0.10	0.045
	TBS value	1.25 (0.12)	1.26	1.21 (0.15)	1.23	1.26 (0.11)	1.27	0.149

*p* value from Welch test to compare the distributions between males and females. DXA = Dual Energy X-ray Absorptiometry; TBS = trabecular bone score; L1-4 = lumbar vertebral body 1-4. The T-score/Z-score indicates how much the measured bone density deviates from the bone density of young, healthy adults/of the corresponding age group.

**Table 4:** Association of DXA T-scores, TBS, vitamin D, and the bone remodeling markers in univariate and multivariate analyses.

		T-Score L1–L4			T-Score Femoral Neck			T-Score Total Femur		
		Beta (95% CI)	*p*-Value	R^2^	Beta (95% CI)	*p*-Value	R^2^	Beta (95% CI)	*p*-Value	R^2^
Univariate	Bone alkaline phosphatase	−0.12 (−0.25, 0.02)	0.093	0.012	−0.02 (−0.14, 0.10)	0.758	0.000	−0.09 (−0.22, 0.04)	0.186	0.008
	25-OH-vitamin D	0.01 (−0.09, 0.11)	0.837	0.000	−0.04 (−0.13, 0.05)	0.376	0.002	0.03 (−0.08, 0.13)	0.587	0.001
	Deoxypyridinoline	−0.01 (−0.18, 0.17)	0.943	0.000	−0.08 (−0.19, 0.04)	0.210	0.006	−0.08 (−0.25, 0.10)	0.374	0.006
Multivariable	Bone alkaline phosphatase	−0.05 (−0.20, 0.10)	0.480		−0.03 (−0.18, 0.12)	0.682		−0.12 (−0.28 0.04)	0.142	
	25-OH-vitamin D	0.03 (−0.06, 0.12)	0.532	0.005	−0.01 (−0.07, 0.04)	0.595	0.003	0.08 (0.04, 0.13)	<0.001	0.031
	Deoxypyridinoline	0.04 (−0.07, 0.15)	0.504		−0.04 (−0.12, 0.04)	0.332		−0.03 (−0.14, 0.08)	0.600	
Univariate	TBS T-score	0.31 (0.19, 0.44)	<0.001	0.095	0.37 (0.25, 0.48)	<0.001	0.130	0.38 (0.26, 0.50)	<0.001	0.139
	TBS Z-score	0.29 (0.14, 0.43)	<0.001	0.079	0.24 (0.12, 0.37)	<0.001	0.070	0.26 (0.13, 0.39)	<0.001	0.075
	TBS value	0.32 (0.20, 0.45)	<0.001	0.101	0.37 (0.26, 0.48)	<0.001	0.135	0.37 (0.25, 0.49)	<0.001	0.133
Multivariable	TBS T-score	0.26 (0.05, 0.47)	0.018	0.068	0.36 (0.16, 0.55)	0.001	0.119	0.37 (0.19, 0.55)	<0.001	0.158
	Bone alkaline phosphatase	−0.03 (−0.17, 0.11)	0.693		0.04 (−0.10, 0.19)	0.545		−0.03 (−0.17, 0.10)	0.645	
	25-OH-vitamin D	0.06 (−0.03, 0.16)	0.204		0.03 (−0.02, 0.08)	0.192		0.13 (0.09, 0.18)	<0.001	
	Deoxypyridinoline	0.06 (−0.04, 0.17)	0.215		−0.01 (−0.09, 0.08)	0.850		0.01 (−0.10, 0.11)	0.876	
Multivariable	TBS Z-score	0.23 (−0.03, 0.48)	0.079	0.052	0.18 (−0.04, 0.41)	0.115	0.035	0.22 (−0.01, 0.44)	0.058	0.083
	Bone alkaline phosphatase	−0.03 (−0.18, 0.12)	0.709		0.00 (−0.14, 0.14)	0.978		−0.07 (−0.22, 0.07)	0.327	
	25-OH-vitamin D	0.05 (−0.06, 0.15)	0.370		0.02 (−0.03, 0.06)	0.521		0.11 (0.06, 0.16)	<0.001	
	Deoxypyridinoline	0.07 (−0.02, 0.17)	0.141		0.01 (−0.07, 0.10)	0.761		0.02 (−0.06, 0.11)	0.594	
Multivariable	TBS value	0.31 (0.11, 0.52)	0.003	0.094	0.40 (0.20, 0.60)	<0.001	0.138	0.40 (0.21, 0.58)	<0.001	0.164
	Bone alkaline phosphatase	−0.03 (−0.17, 0.11)	0.642		0.04 (−0.11, 0.18)	0.623		−0.04 (−0.18, 0.09)	0.530	
	25-OH-vitamin D	0.07 (−0.03, 0.16)	0.161		0.04 (−0.01, 0.09)	0.150		0.13 (0.09, 0.18)	<0.001	
	Deoxypyridinoline	0.07 (−0.03, 0.17)	0.195		−0.01 (−0.09, 0.07)	0.813		0.00 (−0.10, 0.11)	0.926	

Beta = standardized regression coefficient, CI = confidence interval, R^2^ = coefficient of determination. 25-OH-vitamin D = 25-hydroxy-vitamin D

## Data Availability

Not applicable.
